# Salvage Therapy Against Infections of MDR *Acinetobacter baumannii* Achieved by Synergistic Effect of Colistin-Containing Therapies—Preliminary Study

**DOI:** 10.3390/microorganisms13061206

**Published:** 2025-05-25

**Authors:** Paweł Kmiecikowski, Aniela Gabriel, Dagmara Depka, Tomasz Bogiel

**Affiliations:** 1 Microbiology Student Science Club, Ludwik Rydygier Collegium Medicum in Bydgoszcz, Nicolaus Copernicus University in Toruń, 9 Maria Skłodowska-Curie Street, 85-094 Bydgoszcz, Poland; kmiecikowski.pawel@gmail.com (P.K.); aniela.gabriel@wp.pl (A.G.); 2 Microbiology Department, Ludwik Rydygier Collegium Medicum in Bydgoszcz, Nicolaus Copernicus University in Toruń, 9 Maria Skłodowska-Curie Street, 85-094 Bydgoszcz, Poland; dagmaradepka@cm.umk.pl; 3 Clinical Microbiology Laboratory, Dr. Antoni Jurasz University Hospital No. 1 in Bydgoszcz, 9 Maria Skłodowska-Curie Street, 85-094 Bydgoszcz, Poland; 4 Department of Propaedeutics of Medicine and Infection Prevention, Ludwik Rydygier Collegium Medicum in Bydgoszcz, Nicolaus Copernicus University, 9 Maria Skłodowska-Curie Street, 85-094 Bydgoszcz, Poland

**Keywords:** *Acinetobacter baumannii*, ampicillin/sulbactam, antibiotics synergy, colistin, multi-drug resistance

## Abstract

Infections caused by multi-drug-resistant *Acinetobacter baumannii* are a global threat. The World Health Organization has recognized carbapenem-resistant *A. baumannii* as critical pathogens for which further research and development of effective drugs are needed. The aim of this study was to identify antibiotic combinations with possible potential for additive or synergistic action with colistin, and thus to find new therapeutic possibilities for the treatment of infections caused by multi-drug-resistant *A. baumannii*. The research involved the two multi-drug-resistant *A. baumannii* strains isolated from hospitalized patients. In this study, six antibiotics were chosen to combine with colistin: amikacin, gentamicin, ampicillin/sulbactam, tigecycline, imipenem, and meropenem. For both strains, the synergistic activity of colistin and ampicillin/sulbactam was demonstrated, and additive activity for ABA25, colistin, and meropenem or imipenem. The MICs of antibiotics that showed synergism with colistin were reduced by 8- to 128-fold. Additive interactions have been shown in colistin combination with carbapenems, aminoglycosides, and tigecycline. The results prove the synergistic effect of the tested antibiotics, which may be helpful in the selection of potentially effective multi-drug therapies and their application in clinical practice, which may involve reducing the doses of colistin in therapy and its toxicity.

## 1. Introduction

Antibiotic resistance can be defined as the ability of bacteria to tolerate the presence of an antibiotic in the environment or simply reduce its activity by undertaking a number of different activities to achieve this goal. This phenomenon emerged long before Fleming’s discovery of antibiotics as a defense mechanism against metabolites of other organisms [1]. Currently, many types of antibiotic resistance mechanisms are known.

The dynamic development of antibiotic resistance over the past few decades has become a growing worldwide threat to the health and life of the entire population. The overuse of antibiotics, in both clinical practice and in livestock farming, has a significant impact on the accelerated development of resistance [2]. Effective therapy for infections caused by multi-drug-resistant (MDR) bacteria increasingly requires the use as a last resort drugs. The problem has become serious enough for the World Health Organization (WHO) to list it as one of the top ten public health threats for 2019 [3].

The long process of introducing new drugs to the pharmaceutical market, and in fact to clinical practice, and the need to often use them as last-resort drugs have a negative impact on the effectiveness of the treatment of bacterial infections. Between 2017 and 2022, only twelve new antimicrobial agents have been approved, but almost all of the listed compounds belong to already known antibiotic groups. It is worth emphasizing that due to the same mechanism of action, a significant part of the new drugs will soon become inactive due to the already existing bacterial resistance. Moreover, the new antibiotics generate resistance in a brief period of time, sometimes even 2–3 years after their introduction to the market, which is often less time than their introduction to general use and makes it necessary to use them as drugs of last resort [4].

Colistin (also known as polymyxin E) is an antibiotic from the polymyxin group that has several mechanisms of action. They lead to lysis of the inner membrane, leakage of periplasmic and cytoplasmic contents, and ultimately to cell death [5]. Shortly after colistin’s introduction to the healthcare system, its use was restricted due to the development of serious side effects, such as nephrotoxicity and neurotoxicity. As a result of growing resistance, colistin was soon re-introduced into wider circulation as a drug of last resort [6]. Colistin is currently used in the treatment of infections caused by carbapenemase-producing bacteria (including *Acinetobacter baumannii*, *Pseudomonas aeruginosa*, *Klebsiella pneumoniae*, etc.); however, due to the dynamic development of antibiotic resistance and high toxicity, particular emphasis is currently placed on the search for alternative therapies, including those using colistin [7].

The currently preferred form of therapy for infection caused by colistin-resistant *A. baumannii* (CoR-AB) is sulbactam/durlobactam, but its low availability is a significant limitation of its use. The backbone for the treatment of these infections is usually ampicillin/sulbactam, with the latter as an active component [8].

Therefore, it is indicated to seek possible synergistic or additive combinations of colistin with antibiotics with a different mechanism of action. There is evidence of the possibility to reduce the minimal inhibitory concentration (MIC) value and even break the resistance barrier of a given strain to combined antibiotics through the synergistic or additive effects of these antibiotics applied in a mixture [9,10]. Two- or three-drug therapy also shows a reduced risk of generating resistance de novo. Moreover, the use of combination therapy makes it possible to reduce the doses of drugs used, thereby reducing the risk of side effects, without affecting the effectiveness of treatment negatively [11].

Among the bacteria exhibiting extremely high antibiotic resistance are Gram-negative rods, particularly *A. baumannii*, which are listed among the most common causes of hospital infections, the so-called ESKAPE pathogens. ESKAPE is a group of six virulent and resistant pathogens, including species such as *Enterococcus faecium*, *Staphylococcus aureus*, *K. pneumoniae*, *A. baumannii*, *P. aeruginosa*, and *Enterobacter* spp.

Carbapenem-resistant *A. baumannii* (CRAB) is the fourth most common cause of death associated with bacterial antibiotic resistance [12]. The bacteria belonging to the *A. baumannii* complex can cause, among others, pneumonia, bacteremia, and urinary tract infections. Patients in intensive care units (ICUs) and those undergoing long-term hospitalization are at particular risk [13,14]. As the problem of antibiotic resistance affects a broad group of species and, therefore, infected patients, the search for innovative therapeutic options can make a real difference in the clinical conditions of many patients

This article examines the checkerboard method to investigate the additive or synergistic activity of the most relevant combinations of antimicrobials with colistin that are in use as monotherapy in available treatment regimens for *A. baumannii* infections. Among the drugs studied are, amikacin, gentamicin, ampicillin/sulbactam, tigecycline, imipenem, and meropenem, which are widely available in clinical practice. To determine synergistic and additive effects, the checkerboard method was used, which is the most convenient and applicable method that can be introduced into everyday laboratory practice. For this purpose, two selected extremely drug-resistant strains of *A. baumannii* from the collection of the Department of Microbiology, Collegium Medicum in Bydgoszcz, Nicolaus Copernicus University in Toruń were chosen. The aim of this study was to identify antibiotic combinations with possible potential for additive or synergistic action with colistin and, thus, to find new therapeutic possibilities for the treatment of infections caused by multi-drug-resistant *A. baumannii*.

## 2. Materials and Methods

### 2.1. Study Design

The research aimed to assess the additive and synergistic combinations of colistin and other antibiotics with a different mechanism of action. The research involved the use of resources from *A. baumannii* strains provided by the Department of Microbiology of the Faculty of Pharmacy of the Nicolaus Copernicus University Collegium Medicum (NCU CM) and the Department of Clinical Microbiology of the dr Antoni Jurasz University Hospital No. 1 in Bydgoszcz, Poland. The criterion for selecting the bacterial strain was the widest possible range of resistance to available antibiotics. Based on the results of a pilot study, two extremely drug-resistant clinical *A. baumannii* strains (named ABA25 and ABA34) were selected. According to previously performed antimicrobial susceptibility testing, both strains were categorized as multi-drug resistant.

### 2.2. Bacterial Isolates and Antimicrobial

The applied strains were derived from two separate patients of the Anesthesiology and Intensive Care Unit of dr Antoni Jurasz University Hospital No. 1 in Bydgoszcz, Poland. Before their use, the strains were frozen at −80 °C (Thermo Fisher Scientific, Waltham, MA, USA) in a Brain–Heart Infusion Broth (Oxoid, Altrincham, Cheshire, UK) with 10% glycerol (Polskie Odczynniki Chemiczne S.A., Gliwice, Poland). Before the initiation of the study, the strains were passaged twice: first, streaked on Columbia Agar supplemented with 5% sheep blood (*bio*Mérieux, Marcy-l’Étoile, France) and incubated at 37 °C for 24 h, and then, on MacConkey Agar (Becton Dickinson, Franklin Lakes, NJ, USA) and incubated in the same conditions. Bacteria obtained from a second passage were used for further assays.

Species identificationwas carried out using a mass spectrometry analyzer—MALDI-TOF MS Biotyper software version 4.2.28 (Bruker, Billerica, MA, USA) with all the strains’ identification procedures performed according to the manufacturers’ instructions. For this purpose, the colonies of individual bacterial strains were placed on each spot of a dedicated plate. Then, one microliter of the reaction matrix was added to each field. After the drying step, the plate was placed in the analyzer, which identifies and classifies the bacteria to the species level, based on the obtained ribosomal protein spectra.

The susceptibility of the tested strains was performed using the BD Phoenix™ automated identification and susceptibility testing system (Becton-Dickinson, Franklin Lakes, NJ, USA) and the Becton-Dickinson Phoenix™ panels NMIC-402 (Becton-Dickinson, Franklin Lakes, NJ, USA).

For the antimicrobial synergistic activity assessment in this study, six antibiotics were chosen to combine with colistin: amikacin, gentamicin, ampicillin/sulbactam, tigecycline, imipenem, and meropenem. The number of possible combinations of different antibiotics with different concentrations reached a total of 576.

All the strains, reagents, and antibiotics were stored according to the manufacturers’ recommendations. The experimental phase of this study was carried out at the Department of Microbiology of NCU CM in Bydgoszcz, Poland.

The strains applied in this study were clinical isolates obtained from patient samples collected for diagnostic purposes as part of routine microbiological diagnostics at University Hospital No. 1 in Bydgoszcz, Poland. Therefore, no additional patient consent was required for their use in this research. All the applied procedures were conducted in accordance with Polish law, university and hospital policies, and the guidelines of the Bioethics Committee of NCU CM in Bydgoszcz.

### 2.3. MIC Determination

The first stage of the study included the determination of the MIC values for each of the tested antibiotics separately by the broth microdilution method using a 96-well polystyrene plate [15]. The applied antibiotics were prepared in a 2-fold concentration dilution series from 0.125 to 256 μg/mL. Each tested antibiotic was assigned to one of the seven horizontal rows A–G (H row was left and served as a positive growth control in an antimicrobial-lacking medium) and labeled appropriately.

### 2.4. Checkerboard Method—Evaluation of Activity for Combinations of Antibiotics

The broth microdilution method was used to evaluate an antibacterial activity of antibiotic combinations, arranging colistin and six antibiotics in a checkerboard format in 96-well plates (as described above) [16]. A series of increasing concentrations was performed from 0.125 μg/mL to 256 μg/mL for colistin and from 0.5 μg/mL to 64 μg/mL for the other drug.

To examine the results for one strain, six 96-well plates were used—one for each antibiotic combination. First, a microdilution series for colistin was created. Aliquots of 50 μL of MHB (Thermo Scientific, Waltham, MA, USA) were added to each well. Subsequently, 50 µL of colistin at a concentration of 1024 μg/mL was added to well number 1 in the corresponding row A–H. Using an automatic multi-channel pipette, 50 μL was transferred from wells in row no. 1 to wells in row no. 2, from no. 2 to no. 3, etc. The last 50 μL aliquots from the wells in row no. 12 were removed.

A microdilution series was created for the remaining antibiotics in Eppendorf tubes. Eight series were prepared for each antibiotic and designed sequentially, using meropenem as an example, from MemA to MemH. A volume of 1 mL of MHB (Thermo Scientific) was added to each tube. Next, 333 μL of 1024 μg/mL antibiotic was added to MemA. Further portions of 650 μL were transferred from MemA to MemB, from MemB to MemC, etc. The last 650 μL from MemH was discarded.

Afterwards, 50 μL of aliquot was taken from MemA to each well in row A, 50 μL from MemB to each well in row B, etc. Subsequently, 50 μL of MHB and 50 μL of bacterial suspension were added to each well at the plate to reach both of the final antimicrobial concentrations.

### 2.5. Fractional Inhibitory Concentration Index

To analyze the obtained results of combining of colistin with antibiotics, with a different mechanism of action, the fractional inhibitory concentration index (FICI) was determined using the formula below:

FICI = FIC_X_ + FIC_Y_ (1)

FIC_X_ = MIC_XC_/MIC_X_ (2)

FIC_Y_ = MIC_YC_/MIC_Y_ (3)

where FIC_X_ means fractional inhibitory concentration for antibiotic X, FIC_Y_ means fractional inhibitory concentration for antibiotic Y, MIC_X_ means minimal inhibitory concentration for antibiotic X used alone, MIC_Y_ means minimal inhibitory concentration for antibiotic Y used alone, MIC_XC_ means minimal inhibitory concentration for antibiotic X in combination, and MIC_YC_ means minimal inhibitory concentration for antibiotic Y in combination [11].

The FICI value allows the effect of a combination of antibiotics to be assessed as antagonistic for FICI > 4, neutral for 1 < FICI ≤ 4, additive for 0.5 < FICI ≤ 1, or synergistic if FICI ≤ 0.5.

## 3. Results

### 3.1. Minimal Inhibitory Concentrations of the Applied Antimicrobials

The MICs of every used antibiotic were determined by the broth microdilution method. Both of the used strains were susceptible to amikacin and gentamicin and resistant to imipenem, meropenem, and colistin (Table 1). Although, according to the EUCAST Recommendations, there is insufficient evidence that *A. baumannii* is a good target for therapy (lack of possibility to assign particular MIC values to a particular resistance phenotype) with tigecycline and ampicillin/sulbactam. Nevertheless, based on the relevance of the available literature, these antimicrobials were included in the study [17].

**Figure 1:**
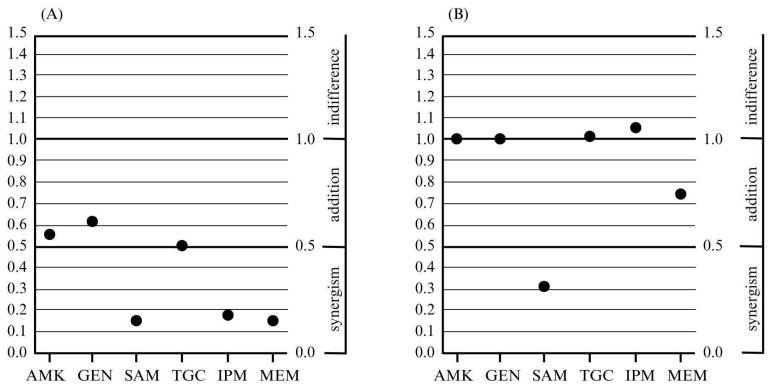
The lowest fractional inhibitory concentration index values obtained for combinations of colistin and other antimicrobials with a different mechanism of action, and the obtained effects of the resulting combination for the tested strains: ABA25 (**A**) and ABA34 (**B**); AMK—amikacin, GEN—gentamicin, SAM—ampicillin/sulbactam, TGC—tigecycline, IPM—imipenem, and MEM—meropenem.

For the ABA25 strain, at the very least, an additive activity was demonstrated for each tested combination. In turn, a synergistic effect was observed in combinations of colistin with imipenem, meropenem, and ampicillin/sulbactam (Table 2, Figure 1).

For the second strain, ABA34, an additive activity was demonstrated for four tested combinations of antibiotics with colistin: amikacin, gentamicin, ampicillin/sulbactam, and meropenem. Meanwhile, a synergistic effect was observed only for the colistin combination with ampicillin/sulbactam (Table 3, Figure 1).

### 3.2. Changes in MIC Values

The MIC of colistin used alone for strain ABA25 was 128 μg/mL, ampicillin/sulbactam—16 μg/mL, imipenem—32 μg/mL, and meropenem—128 μg/mL. The MIC of colistin in combinations providing antibiotic synergism decreased up to 128-fold, the MIC of ampicillin/sulbactam or imipenem decreased up to 16-fold, while the MIC of meropenem—64-fold (Table 4).

The MIC of colistin used alone for strain ABA34 was 8 μg/mL, and ampicillin/sulbactam—32 μg/mL. The MIC of colistin in combination with ampicillin/sulbactam, which provided antibiotic synergy, was reduced by up to 8-fold and the MIC of ampicillin/sulbactam by 16-fold (Table 5).

## 4. Discussion

The previous studies indicate good effects of combined antibiotic therapy for infections with multi-drug-resistant bacteria, which coincides with the results obtained in this research [8,9,10,18,19,20,21,22,23,24,25,26]. The aim of this study was to identify antibiotic combinations that provide additive or synergistic effects with colistin, and thus find new possible therapeutic options for the treatment of infections caused by multi-drug-resistant *A. baumannii* (MDR-AB). The checkerboard assay performed in this study indicated the presence of the additive and synergistic effects of a few combinations of antibiotics against a multi-drug-resistant *A. baumannii* strain.

In this study, synergism was demonstrated for the combinations of colistin with imipenem, meropenem, and (for both strains) ampicillin/sulbactam. Addition was demonstrated for all antibiotics combinations. For colistin with amikacin, gentamicin, ampicillin/sulbactam, and meropenem its was demonstrated for both tested strains. In the meta-analysis of fifty-six combination therapy studies, Scudeller et al. showed partially consistent results with the present study. The time–kill test demonstrated synergism for, among others, the combinations of colistin and meropenem and colistin and imipenem. Different results were shown for colistin and tigecycline (synergism was observed) and for colistin and ampicillin with sulbactam (no synergism was observed, but this combination was tested only on two *A. baumannii* strains) [27]. Consistent with the results of the present study is the analysis of eighty-four studies (covering eight hundred and eighteen *A. baumannii* strains) by Karakonstantis et al., according to which tigecycline-based combinations are rarely synergistic against antibiotic-resistant *A. baumannii* strains at clinically achievable antibiotic concentrations [19].

There is probably no universal antibiotic combination that will show additive or synergistic activity against all *A. baumannii* strains [19]. Therefore, future studies should pay attention to the choice of drug combinations studied. In this study, combinations with colistin were selected. Since polymyxins are the basis of therapy for infections caused by *Acinetobacter* species, an exploration of colistin combinations with other antibiotics appears to be a promising research direction [28]. However, one should not forget about combinations with other antibiotics, e.g., polymyxin B [24,27,29,30] or ampicillin/sulbactam [10,31], which both have promising potential for synergistic activity against *A. baumannii*. There are also studies indicating decreased mortality while using sulbactam-based regimens, which may be worth more attention [32].

Very important is the evidence that a combination of antibiotics can overcome the natural resistance of bacteria to one of them. Mantzana et al. exposed thirty clinical isolates of *A. baumannii* resistant to carbapenems and colistin to the combination of colistin and daptomycin, achieving a synergistic effect in 90%. Of the eighty isolates treated with the combination of colistin and rifampicin, a synergistic effect was demonstrated in 93.75% of the strains [21]. In another study performed by Mantzana et al., two hundred *A. baumannii* strains resistant to carbapenems and colistin were examined. Synergism was observed for the combination of colistin and rifampicin in 91.52% of the tested strains, while for colistin and daptomycin in 100% [22]. In this study, no antibiotic that did not include *A. baumannii* in its spectrum of activity was used. It is worth using such antibiotics in future studies, because MDR-AB has not yet developed acquired resistance mechanisms to them.

This study showed a significant decrease in the MICs of antibiotics used in combination compared to their use in monotherapy. The MIC of colistin decreased 8-, 32-, 64-, and 128-fold, and the MICs of the remaining antibiotics in combination with colistin decreased 16- or 64-fold. It is important that such a reduction in drug sensitivity may be very beneficial, so the focus should not always be solely on a synergistic or additive effect. Rodjun et al., testing the combination of colistin with sitafloxacin against MDR-AB, CRAB, and CoR-AB, obtained synergism levels of 3.4%, 3.1%, and 20.9%, respectively, but as many as 95.4% of CoR-AB isolates acquired susceptibility to colistin [33]. In this way, multi-drug therapy may allow for a reduction in antibiotic doses and thus a reduction in adverse effects without negatively affecting the treatment outcome. In the case of colistin therapy, it is particularly recommended to reduce the dose due to its high toxicity [34].

Some limitations of the conducted research should be noted. First, only two bacterial strains were examined, which is not a representative group to generalize the results to other strains. However, two different clinical strains were selected with exceptional drug resistance, which became a representative model for assessing really difficult cases of infections. The second limitation is that only one method was used. For the results to be more reliable, it would be necessary to repeat the antibiotic synergism assay on the same strains using a different method, such as a time–kill test, which is considered asthe gold standard [35]. This would also allow for a comparison of these two methods for determining antibiotic synergistic activity. However, a methodology was chosen that is relatively easy to perform and, in the end, applicable in routine diagnostic schemes in microbiology laboratories worldwide. In addition, this study included a relatively small number of antibiotics, which does not allow for a full assessment of possible interactions between individual antibacterial agents. The limitations of the study pointed out above indicate the need for further, more comprehensive studies that would include a greater variety of bacterial strains, a variety of analysis methods, and a wider range of antimicrobials tested.

While the current study focused on certain combinations of only two antibiotics, exploring triple combinations of antimicrobials could also be beneficial, especially since adding a third one to a pair that has not shown activity may result in a synergistic effect [20]. Several drugs in combination may be particularly advantageous in overcoming resistance mechanisms. Further research is needed so that antibiotic synergism, which can overcome antibiotic resistance in bacteria, is implemented in clinical practice.

Further studies on combined antibiotic therapy should take into account epidemiological differences in bacterial resistance in different regions of the world and some pressing issues related to synergism research. Therefore, it is a necessity to prompt the creation of epidemiological databases on the sensitivity of bacteria to specific antibiotic combinations. However, significant differences in antibiotic resistance profiles of bacterial strains in different regions of Europe and the world should be taken into account. When studying the response of *A. baumannii* isolates to antibiotic combinations, the genetic basis of their antibiotic resistance is sometimes investigated [25,36]. The need to analyze the correlation between the level of response to drug combinations and resistance mechanisms should also be considered, as this could help to better understand antibiotic synergistic and additive effects. The antibiotic synergism described in the results of studies is sometimes demonstrated for concentrations that are not clinically significant [19]. There is also a lack of evidence showing that in vitro synergistic activity correlates with clinical outcomes, and some common antibiotic combinations that had demonstrated therapeutic efficacy in vitro have failed to demonstrate benefits in randomized clinical trials [8]. These data suggest the need to conduct randomized clinical trials of antibiotic combinations with proven in vitro effectiveness. Future research should also aim to identify antibiotic combinations that are effective against bacterial biofilms [37] and to modify the methodology used in order to partially make in vitro conditions similar to in vivo conditions, such as in the polymicrobial checkerboard assay, which can examine polymicrobial populations [38].

## 5. Conclusions

This study demonstrated synergy between the effects of colistin combinations with imipenem, meropenem, and (for both strains) ampicillin/sulbactam. The choice of these combinations for salvage therapy may be a promising finding. An understanding of the mechanisms of interactions between colistin and other drugs can provide crucial insights into developing more effective treatment protocols in the treatment of infections caused by multi-drug-resistant *A. baumannii* and other pathogens. Determining the most optimal dosages for each drug involved is crucial after conducting pharmacokinetic and pharmacodynamic studies. An exploration of a wider variety of antibiotics may result in the discovery of new, active, relatively safe, and non-toxic, attractive antimicrobial combinations.

## Figures and Tables

**Table 1:** Minimal inhibitory concentrations and antibiotic sensitivity of each tested antibiotic for *A. baumannii* strains ABA25 and ABA34.

Antibiotic	ABA25 Strain		ABA34 Strain	
	MIC [μg/mL]	Antibiotic Sensitivity	MIC [μg/mL]	Antibiotic Sensitivity
amikacin	1	sensitive	1	sensitive
gentamicin	4	sensitive	4	sensitive
ampicillin/sulbactam	16	-	32	-
tigecycline	2	-	1	-
imipenem	32	resistant	32	resistant
meropenem	128	resistant	256	resistant
colistin	128	resistant	8	resistant

**Table 2:** The number and percent of antagonistic, indifferent, additive, and synergistic tested combinations and the lowest FICI value obtained for strain ABA25 (FICI—fractional inhibitory concentration index).

Drug in Combination with Colistin	Number (%) of Combinations				Lowest FICI
	Antagonistic	Indifferent	Additive	Synergistic	
amikacin	60 (62.5%)	26 (27.08%)	4 (4.17%)	0 (0%)	0.5625
gentamicin	36 (37.5%)	30 (31.25%)	4 (4.17%)	0 (0%)	0.6250
ampicillin/sulbactam	12 (12.5%)	33 (34.38%)	13 (13.54%)	12 (12.5%)	0.1563
tigecycline	48 (50%)	27 (28.13%)	9 (9.38%)	0 (0%)	0.5020
imipenem	0 (0%)	36 (37.5%)	12 (12.5%)	8 (8.33%)	0.1875
meropenem	0 (0%)	16 (16.67%)	14 (14.58%)	14 (14.58%)	0.1563

**Table 3:** The number and percentage of antagonistic, indifferent, additive, and synergistic tested combinations and the lowest FICI value obtained for strain ABA34 (FICI—fractional inhibitory concentration index).

Drug in Combination with Colistin	Number (%) of Combinations				Lowest FICI
	Antagonistic	Indifferent	Additive	Synergistic	
amikacin	72 (75%)	18 (18.75%)	1 (1.04%)	0 (0%)	1.0000
gentamicin	56 (58.33%)	17 (17.71%)	1 (1.04%)	0 (0%)	1.0000
ampicillin/sulbactam	32 (33.33%)	25 (26.04%)	6 (6.25%)	4 (4.17%)	0.3125
tigecycline	70 (72.92%)	16 (16.67%)	0 (0%)	0 (0%)	1.0160
imipenem	27 (28.13%)	16 (16.67%)	0 (0%)	0 (0%)	1.0630
meropenem	29 (30.21%)	12 (12.5%)	1 (1.04%)	0 (0%)	0.7500

**Table 4:** MIC value changes determined in synergistic antibiotic combinations for the ABA25 strain.

Antibiotics Combination	Colistin MIC Decrease [μg/mL]	Other Antibiotic MIC Decrease [μg/mL]
colistin + ampicillin/sulbactam	128 → 1/(128-fold)	16 → 1/(16-fold)
colistin + imipenem	128 → 4/(32-fold)	32 → 2/(16-fold)
colistin + meropenem	128 → 2/(64-fold)	128 → 2/(64-fold)

**Table 5:** Change in MIC value determined in the synergistic antibiotic combination for strain ABA34.

Antibiotics Combination	Colistin MIC Decrease [μg/mL]	Other Antibiotic MIC Decrease [μg/mL]
colistin + ampicillin/sulbactam	8 → 1/(8-fold)	32 → 2/(16-fold)

## Data Availability

The original contributions presented in this study are included in the article. Further inquiries can be directed to the corresponding author.

## Abbreviations

The following abbreviations are used in this manuscript:

CoR-AB	colistin-resistant *A. baumannii*
CRAB	carbapenem-resistant *A. baumannii*
FICI	Fractional Inhibitory Concentration Index
MDR	Multi-drug resistant
MDR-AB	Multi-drug-resistant *A. baumannii*
MHB	Mueller Hinton Broth
MIC	minimal inhibitory concentration
NCU CM	Nicolaus Copernicus University Collegium Medicum
WHO	World Health Organization
